# 350. Clinical impact of positive fungal blood cultures for diagnosis and treatment of fungal infections.

**DOI:** 10.1093/ofid/ofac492.428

**Published:** 2022-12-15

**Authors:** Miguel A Chavez, Patrick R Ching, Andrej Spec, Kevin Hsueh

**Affiliations:** Washington University in St. Louis, St. Louis, Missouri; Washington University in St. Louis, St. Louis, Missouri; Washington University in St. Louis, St. Louis, Missouri; Washington University in St. Louis, St. Louis, Missouri

## Abstract

**Background:**

Fungal blood cultures are usually ordered when sepsis secondary to disseminated fungal infection is suspected. We aimed to analyze whether positive fungal blood cultures had an added clinical impact over other conventional microbiological tests.

**Methods:**

We performed a retrospective study of all patients for whom fungal blood cultures were performed for any indication at our institution from June 2018 to March 2022. We reviewed informatics database and medical records to assess the microbiological and clinical impact of positive fungal blood cultures during the initial admission. Assessment of clinical impact was analyzed using a list of predefined positive, negative, and no impact scenarios (Table 1) based by the treating team’s decisions.

**Results:**

In total, 4447 fungal blood cultures were performed in 3648 admissions during our study period. The overall positivity rate of fungal blood cultures was 6.4% (n=284), of which only 130 (2.9%) were positive for fungi. The most common isolated fungi were *Candida* spp. (71, 55%), followed by *Histoplasma* spp. (16, 12%), *Cryptococcus* spp. (12, 9%), and unidentified mold (9, 7%) (Table 1). The median time to positivity was 106 hours (IQR 79-177). Only 21 (16%) fungal blood cultures resulted in a change in management. Positive fungal blood cultures for fungi led to a positive clinical impact in 17 (13%), negative clinical impact in 4 (3%), and no clinical impact in 109 (84%). Most fungal blood cultures confirmed other conventional microbiological diagnosis (71, 55%) or result was not acted upon (26, 20%). Organisms where fungal blood culture had a positive impact included *C. albicans* (n=4), and *C. parapsilosis* (n=3), while *Cladosporium* spp. (n=2) was the most frequent fungi with a negative clinical impact. Isolated fungi that had no clinical impact because of other confirmatory tests included *Candida* spp. (n=57), *Histoplasma* spp. (n=13) and *Cryptococcus neoformans* (n=10).

**Table 1.**
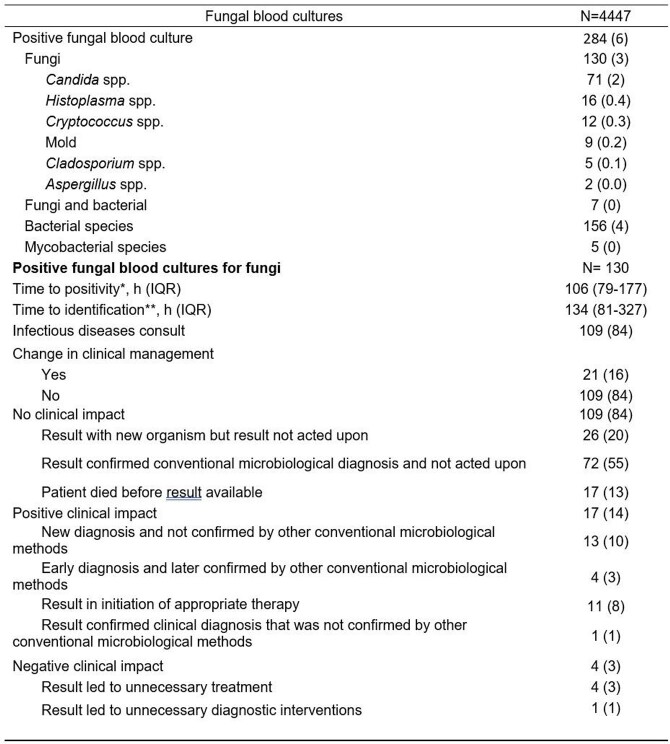
Microbiological characteristics and clinical impact of fungal blood cultures.

**Conclusion:**

Most of positive fungal blood cultures for fungi resulted in no immediate clinical impact due to other microbiological tests available sooner or isolates thought to be non-clinically significant. Further studies should evaluate the long-term impact of fungemia and identify stewardship interventions to optimize clinical utility of fungal blood cultures.

**Disclosures:**

**All Authors**: No reported disclosures.

**Table 1.** Microbiological characteristics and clinical impact of fungal blood cultures.

Abbreviations: h hours; IQR, interquartile range.

*Time to positivity was defined as the time from the fungal blood culture collection until growth and preliminary identification of a fungal organism on staining by the microbiology laboratory. **Time to identification was defined as the time from the fungal blood culture collection until identification of the fungal organism by the microbiology laboratory.

